# New Dimensions of Research on Actinomycetes: Quest for Next Generation Antibiotics

**DOI:** 10.3389/fmicb.2016.01295

**Published:** 2016-08-19

**Authors:** Polpass Arul Jose, Bhavanath Jha

**Affiliations:** ^1^Marine Biotechnology and Ecology Division, CSIR – Central Salt and Marine Chemicals Research InstituteBhavnagar, India; ^2^Academy of Scientific and Innovative Research (AcSIR), Council of Scientific and Industrial ResearchNew Delhi, India

**Keywords:** actinomycetes, natural products, antibiotics, drug discovery, genomics, metabolomics

## Abstract

Starting with the discovery of streptomycin, the promise of natural products research on actinomycetes has been captivating researchers and offered an array of life-saving antibiotics. However, most of the actinomycetes have received a little attention of researchers beyond isolation and activity screening. Noticeable gaps in genomic information and associated biosynthetic potential of actinomycetes are mainly the reasons for this situation, which has led to a decline in the discovery rate of novel antibiotics. Recent insights gained from genome mining have revealed a massive existence of previously unrecognized biosynthetic potential in actinomycetes. Successive developments in next-generation sequencing, genome editing, analytical separation and high-resolution spectroscopic methods have reinvigorated interest on such actinomycetes and opened new avenues for the discovery of natural and natural-inspired antibiotics. This article describes the new dimensions that have driven the ongoing resurgence of research on actinomycetes with historical background since the commencement in 1940, for the attention of worldwide researchers. Coupled with increasing advancement in molecular and analytical tools and techniques, the discovery of next-generation antibiotics could be possible by revisiting the untapped potential of actinomycetes from different natural sources.

## Introduction

Actinomycetes are ubiquitous Gram-positive bacteria that constitute one of the largest bacterial phyla with characteristic filamentous morphology and high G+C DNA. The actinomycetes have been recognized as premier source and inspiration for a substantial fraction of antibiotics that play an important role in human health. The most striking fact is that these filamentous bacteria have evolved with the wealth of biosynthetic gene clusters and thereby show an unprecedented potential in production biologically active natural product scaffolds. However, last two decades has seen a move by pharmaceutical giants away from microbial natural product discovery efforts, and such efforts continue to flourish in research institutes with promising results. The continued research efforts of academic research institutes, with post-genomic technological innovations, rejuvenate natural product research and compose a clarion call to worldwide researchers for tuning into microbial natural products research.

## The Classic Actinomycetes Research

**FIGURE 1:**
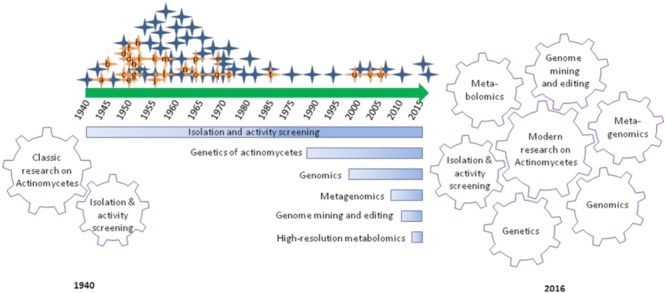
**Graphical summary of research and developments focused on antibiotic discovery from actinomycetes over 76 years.** Hunting of antibiotics from actinomycetes has emanated with the discovery of actinomycin in 1940 (a) and lined up with several commercially important antibiotics and their derivatives: streptomycin (a), cephalosporins (b), Chloramphenicol (c), neomycin (d), tetracycline (e), nystatin (f), virginiamycin (g), erythromycin (h), lincomycin (i), vancomycin (j), noviobiocin (k), rifamycin (l), kanamycin (m), nalidixic acid (n), fusidic acid (o), gentamicin (p), trimethoprim (q), fostomycin (r), ribostamycin (s), mupiriocin (t), linezolid (u), daptomycin (v), and platensimycin (w). Classic actinomycetes research was driven by isolation and activity screening approach. Whereas, modern actinomycetes research is driven by array of breakthroughs in genetics, genomics, metagenomics, genome mining and editing and high-resolution metabolomics, in association with classical approach.

If we look back to about 76 years of actinomycetes research that focused on hunting bioactive metabolites of public welfare, over 5000 compounds have been reported and contributed to the development of 90% of commercial antibiotics being used for either clinical or research needs. In this long course, actinomycetes research evolved several aspects from isolation and activity screening to modern post-genomic secondary metabolites research (**Figure 1**). The first report of streptomycin by Selman Waksman and associates in the 1940s and subsequent development as drug encouraged pharmaceutical companies and researchers to put their large scale efforts on microbial natural products research (Demain and Sanchez, 2009). The efforts were largely depending on the recovery of microorganisms from diverse environmental samples, and screening for the desired bioactivity. The approach brought the golden era (1950–1970) of antibiotic discovery evidenced by the commercialization of several life-saving antibiotics including streptomycin, vancomycin, rifamycin, and so on (Mahajan and Balachandran, 2012). In subsequent decades, the rediscovery of known compounds and technical challenges associated with purification and structure elucidation of new compounds largely declined the classic efforts (Bérdy, 2012). Despite the evidence of a decline in microbial natural products research, continued innovations in sampling and acquisition of potential actinomycetes from previously unexplored sources are being continued by several academic research groups and mitigate risks of the rediscovery of known compounds and augmented availability of diverse actinomycetes that are fundamental matters to the long term actinomycetes research.

## In Progress

Progress is crucial in several aspects of actinomycetes research that includes (1) isolation and dereplication of actinomycete isolates, (2) prediction and identification novel compounds, (3) enhancing production titers of potential compounds, (4) uncovering genome information and associated biosynthetic potential, (5) collection and processing of genomic data, (6) mining, editing and heterologous expression of cryptic gene clusters, and (7) comprehensive metabolic profiling, under a broad spectrum of main areas such as genetics, genomics and metabolomics.

Establishing actinomycete resources is one of the basic requirements for culture-dependent natural products research. To address this, researchers are learning how to cultivate the unexplored actinomycete biodiversity in diverse environments and such efforts have led to cultivation of numerous novel actinomycetes from marine sediments (Becerril-Espinosa et al., 2013), hydrothermal vents (Thornburg et al., 2010), solar salterns (Jose and Jebakumar, 2013), desert soils (Mohammadipanah and Wink, 2016), red soils (Guo et al., 2015), sponges (Sun et al., 2015), insects (Matsui et al., 2012; Kurtböke et al., 2015), and plants (Masand et al., 2015). On the other hand, dereplication of isolated strains has attained a new pitch with gene specific as well as metabolic fingerprinting approaches (Hou et al., 2012; Forner et al., 2013). Collectively, the united success in isolation and dereplication facilitates the prioritization of the isolates which could be cellular factories with the innate biosynthetic capability to produce novel compounds. One such approach has been practiced to isolate 64 distinctive actinomycetes from 12 different marine sponge species, and to prioritize two unique strains that showed anti-trypanosomal activity as well as uniqueness in metabolomic profile and richness of unidentified natural products (Cheng et al., 2015).

Prediction and identification of novel compounds from actinomycetes including those with low production titers have become relatively straight forward through the advent of high-resolution liquid chromatography-mass spectrometry (HR-LC-MS) and allied database search (Tawfike et al., 2013; Doroghazi et al., 2014; Wu et al., 2016). Recently, Wu et al. (2016) were able to demonstrate the employability of NMR-based metabolic profiling method to streamline microbial biotransformation and to determine the best harvesting time of actinomycetes for antibiotic production. Technical breakthroughs also in gene level understanding and recombineering of producer strains provide an attractive choice to improve the production titers of structurally complex natural products by microbial fermentation (Zhang et al., 2016).

Exploring the biology of secondary metabolites production in actinomycetes through genetics has provided a foremost share to our current knowledge. Dramatic and sustained increase in understanding the genetics and enzymology of secondary metabolites biosynthesis in actinomycetes, especially *Streptomyces* throughout the 1990s have also facilitated endurance of natural product search in this admirable bacterial group. As a noteworthy foundation, *S. coelicolor* A3(2) has genetically been recognized as a model for the actinomycetes, and the whole genome was announced with versatile *in vivo* and *in vitro* genetics (Bentley et al., 2002). The genome analysis of *S. coelicolor* A3(2) has revealed the abundance of previously uncharacterized gene clusters, metabolic enzymes, particularly those likely to be involved in the production of natural products. As a latest accomplishment, the marine actinomycete genus *Salinispora* has been established as a robust model organism for natural product research (Jensen et al., 2015). It has remarkable biosynthetic capacities with 17 diverse biosynthetic pathways of which only four had been linked to their respective products.

The genome information of cultured and uncultured actinomycetes is being promptly updated. Over 1304 actinomycetes genome have been reported as on March 2016 and with the advent of molecular genetics and next-generation genome analysis rapid submissions are expected in near future. Analyses of genomes of actinomycetes have revealed that numerous ‘cryptic’ or ‘orphan’ biosynthetic gene clusters with the potential to direct the production of an ample number of novel, structurally diverse natural products (Challis, 2014; Gomez-Escribano et al., 2016). Subsequently, mining of actinomycetes genome has sketched new directions into the ongoing drug discovery efforts. One such approach has been to mine a collection of 10,000 actinomycetes for novel phosphonic acids, and have laid an intriguing foundation for rapid, large-scale discovery of other classes of natural products (Ju et al., 2015).

Improvements made in bioinformatics methods, particularly specific for natural product gene cluster identification and functional prediction aids in the processing of bulk genomic data of actinomycetes (Alam et al., 2011; Doroghazi et al., 2014; Abdelmohsen et al., 2015). However, sufficient insights into the biology and ecology of antibiotic production are needed to understand the precise triggers and cues required to activate silent gene clusters (Abdelmohsen et al., 2015; Kolter and van Wezel, 2016).

As a great breakthrough, the advent of RNA-guided DNA editing technology Clustered Regularly Interspaced Short Palindromic Repeats (CRISPRs)/Cas9 substantially promises for application to genome modification in biosynthetic gene clusters of actinomycetes (Huang et al., 2015). Obviously, this molecular tool can be used in the engineering of non-model native hosts to heterologous production hosts for the biosynthesis of desired natural products. Continued technological and conceptual advances in engineering microbial hosts will open up opportunities to fully explore and harness Nature’s immensely diverse chemical repertoire (Zhang et al., 2016).

## Future Perspectives

Actinomycetes have been recognized as a premier source of biopharmaceuticals especially antibiotics over several decades. Our universe is rich of diverse unexplored and underexplored environments that could be considered for isolation of novel members of actinomycetes. This could amend our actinomycetes repository with a continuous supply of novel biosynthetic gene clusters and natural product scaffolds on which current research reorient on. Continued advances in genomics and metabolomics reserve a next-generation natural products research and unwrap the wider opportunities on the exploitation of actinomycetes that represent an important asset for the discovery of pharmaceutically valuable compounds. The technological and conceptual advances will drive a transition of “searching for desired natural products” to “designing for desired products” from actinomycetes. Through this article, it is evinced that despite an interim decline in actinomycetes research, new avenues are open now and seek the active attention of researchers throughout the world. Those countries well endowed with the natural resources may deem to fund microbial natural products research especially actinomycetes research for extending the inventions of novel antibiotics of industrial significance to triumph the escalating microbial resistance and infectious diseases.

## Author Contributions

All authors listed, have made substantial, direct and intellectual contribution to the work, and approved it for publication.

## Conflict of Interest Statement

The authors declare that the research was conducted in the absence of any commercial or financial relationships that could be construed as a potential conflict of interest.
